# Toward a Behavior Theory–Informed and User-Centered Mobile App for Parents to Prevent Infant Falls: Development and Usability Study

**DOI:** 10.2196/29731

**Published:** 2021-12-20

**Authors:** Nipuna Cooray, Si Louise Sun, Catherine Ho, Susan Adams, Lisa Keay, Natasha Nassar, Julie Brown

**Affiliations:** 1 The George Institute for Global Health Faculty of Medicine and Health UNSW Sydney Newtown Australia; 2 School of Women’s and Children’s Health Faculty of Medicine and Health UNSW Sydney Sydney Australia; 3 Department of Paediatric Surgery Sydney Children's Hospital Randwick Australia; 4 School of Optometry and Vision Science Faculty of Medicine and Health UNSW Sydney Sydney Australia; 5 Children’s Hospital at Westmead Clinical School Faculty of Medicine and Health University of Sydney Sydney Australia

**Keywords:** child injury, Behaviour Change Wheel, mobile app, mobile phone

## Abstract

**Background:**

Falls account for approximately 50% of infant injury hospitalizations, and caretaker behavior is central to preventing infant falls. Behavior theory–informed interventions for injury prevention have been suggested, but to date, few have been reported. The potential of using smartphones for injury prevention intervention delivery is also underexploited.

**Objective:**

This study aims to develop a behavior theory– and evidence-based as well as user-centered digital intervention as a mobile app for parents to prevent infant falls following agile development practices.

**Methods:**

Infant falls while feeding was selected as the fall mechanism to demonstrate the approach being taken to develop this intervention. In phase 1, the Behaviour Change Wheel was used as a theoretical framework supported by a literature review to define intervention components that were then implemented as a mobile app. In phase 2, after the person-based approach, user testing through think-aloud interviews and comprehension assessments were used to refine the content and implementation of the intervention.

**Results:**

The target behaviors identified in phase 1 were adequate rest for the newborn’s mother and safe feeding practices defined as prepare, position, and place. From behavioral determinants and the Behaviour Change Wheel, the behavior change functions selected to achieve these target behaviors were psychological capability, social opportunity, and reflective motivation. The selected behavior change techniques aligned with these functions were providing information on health consequences, using a credible source, instruction on performing each behavior, and social support. The defined intervention was implemented in a draft Android app. In phase 2, 4 rounds of user testing were required to achieve the predefined target comprehension level. The results from the think-aloud interviews were used to refine the intervention content and app features. Overall, the results from phase 2 revealed that users found the information provided to be helpful. Features such as self-tracking and inclusion of the social and environmental aspects of falls prevention were liked by the participants. Important feedback for the successful implementation of the digital intervention was also obtained from the user testing.

**Conclusions:**

To our knowledge, this is the first study to apply the Behaviour Change Wheel to develop a digital intervention for child injury prevention. This study provides a detailed example of evidence-based development of a behavior theory–informed mobile intervention for injury prevention refined using the person-based approach.

## Introduction

### Background

Falls account for almost half of all injury-related hospitalizations in infants aged <1 year [1], with potential lifelong consequences. Infant falls are often explained by the characteristics of natural development (rolling, exploring, and natural curiosity), which occurs rapidly over the first year of life. Falls frequently happen when caretakers are underprepared for risks associated with this rapid motor development and environments are inappropriate or not well matched to the developmental level. The latter includes misuse of nursery furniture. Age-appropriate injury prevention education for caretakers and home safety assessments have therefore been suggested as potential interventions for infant fall prevention in previous studies [2,3], and there is good evidence that parenting interventions can be effective for reducing child injury generally [4]. Although many falls prevention programs target children aged <5 years and there are a few proven interventions effective for preventing child injury in the home generally, there is currently a paucity of proven theory-driven fall prevention interventions specifically targeting caretaker behavior and environmental risks to reduce falls in children aged <1 year [5]. We intend to fill this gap by developing an intervention targeting caretaker behavior and attention to environmental risks to reduce the risk of falls in children aged <1 year.

As fall mechanisms change with the age of the infant [6], any type of intervention needs to account for the different contexts or scenarios related to falls throughout the first year of life. It is well understood that educational interventions alone may not lead people to act on the information they receive; therefore, it is important that the intervention be firmly grounded in behavior change theory such as the one underpinning the Behaviour Change Wheel [7]. This is a commonly used theoretical framework in the design of behavior change interventions targeting a broad array of public health problems [8-11].

Smartphones are an ideal delivery channel for child injury prevention interventions, with new parents increasingly using technology to access health information, especially in countries with high smartphone use [12]. Smartphones and digital technologies and apps also provide a mechanism for delivering a greater array of behavior change techniques targeting behavior change than paper-based or person-to-person intervention delivery methods. They also provide an opportunity for remote engagement with specific sectors of the community when one-to-one engagement is difficult, such as in a pandemic [13,14] or geographically isolated locations. A behavior change intervention combined with mobile technology is known as a digital behavior change intervention (DBCI). Given the flexibility of this delivery mechanism and the growing evidence for the effectiveness of DBCIs in other areas of public health, particularly those DBCIs grounded in behavior theory [15], we plan to develop our intervention as a DBCI.

As usability is critical to the success of DBCIs [16], user testing is an important part of the intervention development process, and think-aloud studies are commonly used for this purpose [17]. Coupled with the Behaviour Change Wheel methodology, this can be used to understand both the hedonic or utilitarian aspects of the DBCI and the appropriateness and anticipated challenges in adherence to embedded behavior change techniques [17]. Information comprehension is another important aspect of usability likely to affect DBCI effectiveness. Although this does not seem to be something routinely assessed in user testing of DBCIs, the need to make sure that the intervention is suitable for users of different levels of literacy has been noted previously [18], and a systematic assessment of comprehension is common in the development of written health information [19].

### Objective

The aim of this study is to develop an intervention using the Behaviour Change Wheel, supported by empirical data and expert feedback, to systematically identify behavior change techniques and implement them digitally (phase 1) and to optimize the digital intervention modules through user feedback and assessment of comprehension of information (phase 2). In previous work, we have identified key fall mechanism priorities [20,21], and following agile development practices [22], we are developing this intervention in a modular way. The key infant fall mechanisms we are targeting in this intervention are falls from furniture, falls when being carried or supported by someone, and falls from baby products. Our approach to developing this multitarget intervention involves the development of 4 distinct modules that address (1) falls from furniture, (2) falls that occur when the baby is feeding, (3) other aspects of home environments where falls occur when the infant is being carried (eg, steps and stairs), and (4) falls from baby products. The same development and user-testing approach is being applied in the development of each of these 4 modules. To allow our development process to be described in detail in a single paper, we have chosen the module targeting infant falls related to feeding as a case study to describe this process.

## Methods

### Two-Phased Approach

**Figure 1:**
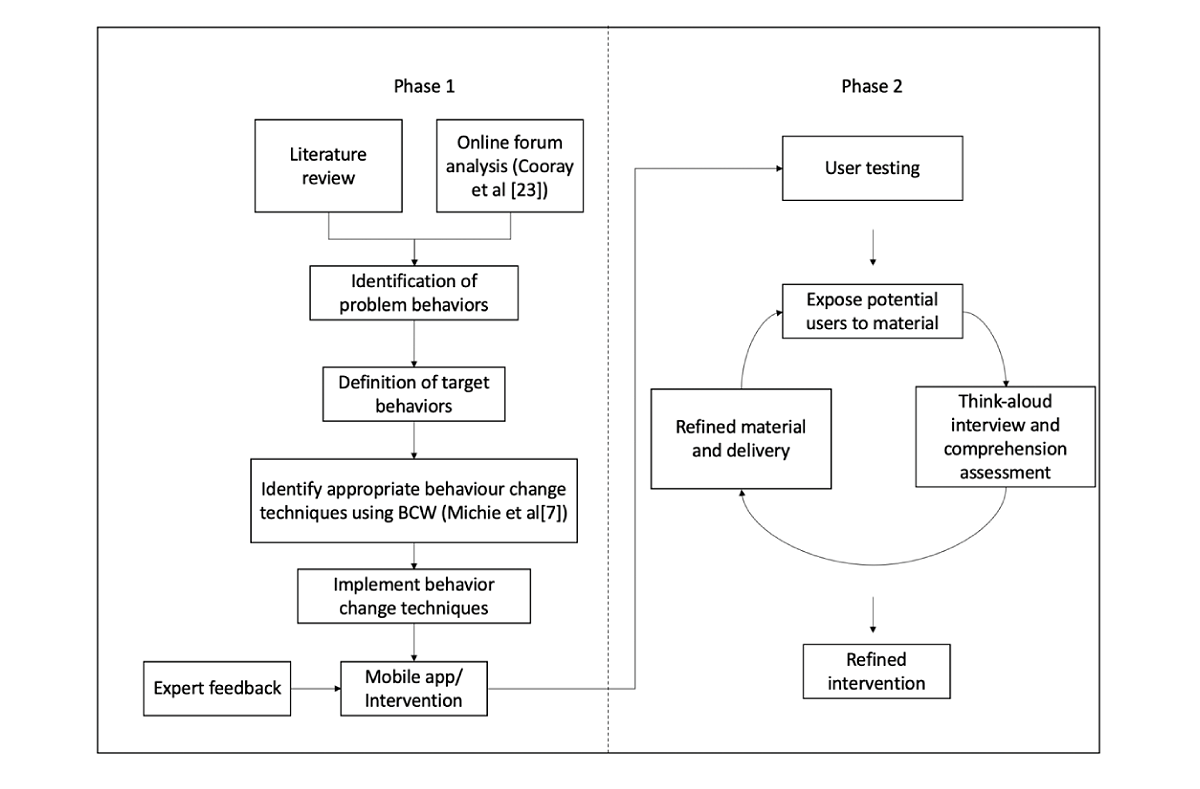
Two-phased development of the intervention. BCW: Behaviour Change Wheel.

Figure 1 depicts the two-phased approach used in developing the intervention module. Phase 1 involved the development and digital implementation of the intervention material, whereas in phase 2, the digital information and delivery method were optimized after think-aloud interviews and comprehension assessment with the target audience.

### Phase 1: Intervention Content Design and Development

The aim of phase 1 is to identify problem and target behaviors to inform and then develop the intervention content for the DBCI. The Behaviour Change Wheel framework [7], a literature review, and a qualitative analysis of infant fall events from a web-based parenting forum [23] were used to identify problem behaviors and target behaviors to inform the intervention strategy. Specifically, problem behaviors were behaviors that would need to change for falls to be prevented. Target behaviors were then chosen if assessed as likely to modify or prevent the *problem behaviors*. The target behaviors were then used in a behavior analysis to identify intervention functions and behavior change techniques following the Behaviour Change Wheel [7] process. In summary, this includes (1) understanding the capability, opportunity, and motivation factors underpinning the target behavior; (2) identifying intervention functions; (3) identifying behavior change techniques to be included; and (4) implementing the selected behavior change techniques in the intervention [7].

The intervention content was then drafted and reviewed by a team of health care professionals, including injury experts, a pediatric surgeon, and content area specialists. They included breastfeeding specialists and midwives. The final draft content was then included in a purpose-built digital intervention module in the form of a mini-app. App feature selection was informed by previous studies reporting common characteristics of health apps to change and manage behaviors [18]. NC conducted the literature review. NC, CH, SA, and JB applied the Behaviour Change Wheel, created the intervention strategy, and developed the intervention content. NC developed the app.

### Phase 2: User Testing and Intervention Optimization

Phase 2 objectives are to ensure usability of the intervention, including comprehension of the intervention content. This was achieved by exposing potential users to the draft intervention content through the mini-app. Ethical approval was granted by the human research ethics committee of South Eastern Sydney Local Health District (2019/ETH00298). Participation involved an initial demographics and falls perception questionnaire, followed by a think-aloud interview as well as a comprehension assessment.

Participants were recruited in sequential rounds of 5 from a single tertiary maternity hospital antenatal ward and day-stay unit. Adult expectant parents were identified as the key user group because the intervention targets fall prevention in infants from birth to 12 months of age and the intention is to ultimately deliver the intervention to this group of the population. To be included, the expectant parents had to be conversant in English and could be first-time or experienced parents. This recruitment method prioritized mothers over fathers; however, this was deemed acceptable for the purposes of this study because mothers are commonly the primary caretakers of infants [24]. Written informed consent was obtained from willing participants.

Participants were individually presented with the mini-app on a study smartphone and asked to provide feedback through a *think-aloud* interview (Multimedia Appendix 1). This interview was audiotaped and analyzed later. The interview involved asking the participants to verbalize their thoughts while they used the digital intervention, after which we administered a set of questions to explore what the participants liked or disliked about the intervention content, along with any suggested improvements. Once completed, the participants were allowed to use the mini-app again, and a structured questionnaire (Multimedia Appendix 2) was used to assess their comprehension of the information provided. This approach has been used in previous studies testing comprehension of medical information [25], as well as by researchers developing consumer materials for child restraint installation [26]. To ensure that all participants were provided with falls prevention information regardless of the state of the mini-app, on completion, they were provided a widely available factsheet detailing advice on childhood falls prevention [27].

The results from the think-aloud interviews and comprehension assessments were analyzed as described in the next section and used to refine the intervention content and mini-app design before the process was repeated on the next round of 5 participants. Iterative rounds of 5 participants with the intervention content and mini-app refinement continued until 80% of the participants demonstrated at least 90% comprehension, which was defined as 4 out of 5 participants in each round achieving a score of at least 11 out of 12 in the comprehension assessment [19].

### Analysis and Refinement

The comments collated from the think-aloud interviews were used in a systematic process of making person-based changes as outlined in Morrison et al [28]. The steps in this process were as follows:

1. Conduct and transcribe the interview
2. Extract negative and positive verbatim comments
3. Tabulate and code comments in a table of potential changes
4. Determine and implement modifications

All discussions were first transcribed verbatim by SLS. The researcher then worked line by line through each transcript to tabulate aspects of the data that showed positive and negative perceptions of the intervention, as well as any suggested modifications. For app refinement, members of the research team considered whether a modification to the intervention program would suitably address the concern expressed in each comment listed in the table. The criteria for making modifications were likely positive impact on drivers of behavior change (capability, opportunity, and motivation) or acceptability and feasibility. If the changes were uncontroversial and feasible to apply, they were implemented immediately. In other cases, more data were collected from another round of testing to seek more opinions before implementing the change. Finally, modifications requiring further tailoring and major changes to the app were discussed with the broad research team and if agreed upon were noted for later implementation in the final integrated app. For analysis of the comprehension questionnaire, comprehension scores were calculated for each user per round of testing, and percentages were tabulated.

## Results

### Phase 1: Intervention Planning and Development

#### Problem Formulation

Table 1 presents a summary of the key themes identified from the literature review and the qualitative analysis of web-based forum discussions [23]. From these themes, the problem behaviors were defined as follows: (1) tired mother falling asleep while feeding her baby (on a chair or on a bed) intentionally or unintentionally and (2) baby left alone on the bed to feed (bottle-feeding) or baby left alone on the bed before or after a feed.

**Table 1:** Key themes identified from the literature review and the qualitative analysis of web-based forum discussions.

Key themes	Support from literature	Scenarios (from web-based parenting forum analysis)
Possibility of sleeping while holding the baby	The possibility of mothers falling asleep while they are feeding their babies [29-33]	“I used to fall asleep while breastfeeding and after nearly dropping Tilly onto a metal table leg I gave up actually breastfeeding at night”
Exhausted mother	During the postpartum period, mothers are often exhausted and tend to fall asleep while feeding their babies [31,34]	“She was about 6 weeks old and I was totally sleep deprived. Sat down on the couch to nurse her, dozed off with her snuggled low in my arms (basically in my lap)...DD rolled down my legs and into the coffee table”
Importance of support and mother calling for help	Interventions should target reducing maternal exhaustion such as implementing mothers’ nap time in the study by Hodges and Gilbert [34]. In addition, mothers need to call for assistance when tired [32]	“...my ex-h had left and I had 3 other children. I was beyond exhausted. More than once I fell asleep while feeding on the couch, only to be woken by my baby crying after she had rolled off me”
Postpartum depression and risk of injury	The evidence of postpartum depression and fall injury relationship [35] and importance of better social support for prevention	—^a^
Parents’ awareness of risk of falls	Parents not aware of the risk of infant falls [34]	“...I fell asleep while feeding and it happened again...but a post on...revealed that it happens to lots of people”
Feeding place and position	Wallace [36] looked at redesigning bed rails of hospital beds. Thus, the target behavior was selected as lying in the middle of the bed when feeding the baby	“...I was breastfeeding him in bed and fell asleep with him on the outside. I woke up when I heard a thud and DS cry”
Risks of cosleeping and always placing the baby in the cot after a feed	Keeping the baby in a separate sleeping place; the best place has been identified as a cot by the mother’s bedside [32,33]	—

^a^Not available.

After further review of the emerging themes listed in Table 1 and discussion with the team of experts, the following target behaviors for intervention development were selected:

1. Getting sufficient rest with the newborn (get help from others, sleep when the baby sleeps, use a breast pump to express milk, and plan sleep)
2. Preparing before the feed
3. Safe positioning during the feed
4. Safe placement of the infant after the feed

Table 2 presents the results of the application of the Behaviour Change Wheel to the identified target behaviors.

**Table 2:** Applying the Behaviour Change Wheel to the identified target behaviors.

COM-B^a^ analysis			Intervention functions		Intervention strategy with BCTs^b^
**Getting enough rest with a newborn**					
	Psychological capability: Knowing ways and techniques to get sufficient rest with a newborn Social opportunity: Getting help from others Reflective motivation: Believing in the importance of getting enough rest for the sake of personal health and baby’s health Automatic motivation: Having the habit of sleeping when the baby sleeps	Education Persuasion Environmental restructuring Enablement		Provide information on the importance of mother getting enough rest for the sake of personal and infant health (BCT: information on health consequences) Provide information on ways to get enough rest with a newborn (BCT: instruction on how to perform the behavior) Inform to discuss sleep arrangements with a support person (BCT: action planning) Inform to use support groups to get better rest (BCT: social support unspecified) All the information is from a credible source (BCT: credible source) Provide reminders to informing to get enough rest (BCT: prompt and cues)	
**Preparing before the feed**					
	Psychological capability: Knowing what is needed for a feed, why it is important to prepare and to prepare before a feed Physical opportunity: Having a feeding basket with prepared items Reflective motivation: Believing in the importance of preparing and understanding the possibility of leaving the infant alone, if unprepared Automatic motivation: Having the habit of preparing before a feed	Education Persuasion Environmental restructuring		Provide information on the importance of preparing and the possibility of leaving the infant alone when unprepared and the risks (BCT: health consequences) Provide information on what is usually needed for a feed and how to prepare before a feed (BCT: instruction on how to perform the behavior) Provide information to prepare a feeding basket and place near the usual feeding position (BCT: adding objects to the environment) Provide a mechanism to ensure self-monitoring behavior (BCT: self-monitoring)	
**Safe positioning during a feed**					
	Psychological capability: Know the consequences and possibility of baby falls while feeding and the common scenarios; know the safe places to feed depending on the situation Reflective motivation: Believing the importance of safe positioning to prevent falls and SUDI^c^	Education Persuasion Training		Provide information on the risk of infant falls when feeding, especially if it involves a risky place or posture, for example, falling asleep while feeding the baby in a chair (BCT: information on health consequences) Provide information on safe feeding places and posture depending on the situation and ways to feed safely (BCT: information on how to perform the behavior)	
**Safe placement of the infant after a feed**					
	Psychological capability: Know the risk of cosleeping, including risk of falls and other fatal sleep accidents; know the possibility of mother falling asleep during or after a feed; know that the cot in the parents’ room is the safe place for the infant to sleep Physical opportunity: Having a good quality cot Reflective motivation: Intentions to put the infant in the cot	Education Persuasion Training Environment restructuring Enablement		Provide information on the adverse outcomes of cosleeping and why the cot is the safest place for the infant to sleep (BCT: information on health consequences) Provide information about cot standards in Australia and why the cot in the parents’ room is the best place for the infant to sleep (BCT: restructuring the physical environment) Inform to put the baby in the cot after a feed (BCT: information on how to perform the behavior)	

^a^COM-B: capability, opportunity, motivation-behavior.

^b^BCT: behavior change technique.

^c^SUDI: sudden unexpected death in infancy.

#### Implementation of the Planned Intervention Strategy (App Development)

To implement the planned intervention strategy, a minimum viable product mini-app was developed for use on the Android platform. The mini-app had 3 main sections. The *Learn* section included information articles with an interlinked *Action* section that provided a self-monitoring mechanism, including one-time and multitime actions. Multitime actions were intended to support behaviors that require repetition. The *Engage* section included a group chat where users could get social support. This feature was also intended to enhance user engagement with the app. In addition, there was an onboarding section to introduce the app to the users. Users were informed about the option of setting up reminders for the *Actions* without fully implementing this feature in the mini-app for testing. The *Learn, Action,* and *Engage* categories were devised to allow appropriate implementation of selected behavior change techniques and align with approaches commonly used in other digital behavior change apps.

### Phase 2: Intervention Optimization Results

#### Participants

A total of 23 women were recruited for the user-testing exercise; 13% (3/23) withdrew because of time constraints. Of the 20 participants, 15 (75%) were aged 26-35 years, 14 (70%) were nulliparous (70%), 10 (50%) were Australian-born, 12 (60%) were in de facto relationships, 13 (65%) were employed full time, and 15 (75%) were living in apartment buildings. Of the 20 participants, 16 (80%) had attained either a university or Technical and Further Education graduate degree or a postgraduate degree and 9 (45%) had high household income (earning more than Aus $150,000 [US $109,500]; Table 3). Target comprehension levels were achieved in 4 rounds (Table 4).

**Table 3:** Participants’ demographics (N=20).

Demographics			Values, n (%)
Gender (female)			20 (100)
**Age (years)**			
	26-35	15 (75)	
	36-45	5 (25)	
**Parity**			
	Multiparous	6 (30)	
	Nulliparous	14 (70)	
Nationality (Australian)			10 (50)
Language spoken at home (English)			20 (100)
**Household income (Aus $; US $)**			
	20,001-100,000 (14,601.30-73,000)	4 (20)	
	100,001-150,000 (73,001.30-109,500)	4 (20)	
	>150,000 (109,500)	9 (45)	
	Decline to answer	3 (15)	
**Marital status**			
	Married	7 (35)	
	Divorced	0 (0)	
	Separated	0 (0)	
	Single parent	1 (5)	
	In a de facto relationship	12 (60)	
**Education level**			
	Primary school, secondary school, some university, or TAFE^a^ diploma	4 (20)	
	University or TAFE graduate	9 (45)	
	Postgraduate degree	7 (35)	
**Employment status**			
	Unemployed	2 (10)	
	Seasonal or casual employment	0 (0)	
	Part-time employment	3 (15)	
	Full-time employment	13 (65)	
	Student (full time, part time, or correspondence)	0 (0)	
	Not applicable or decline to answer	2 (10)	
**Primary residence**			
	A stand-alone house	2 (10)	
	A semidetached town house or duplex	1 (5)	
	A townhouse complex	2 (10)	
	An apartment building	15 (75)	

^a^TAFE: Technical and Further Education.

**Table 4:** Results of comprehension assessment in each user-testing round (5 participants per round).^a^

	Score (%), mean (SD)	Score (%), range	Participants scoring 90%, n (%)
Round 1 participants’ comprehension scores	84.8 (12.7)	66-100	2 (40)
Round 2 participants’ comprehension scores	80 (19.2)	50-100	2 (40)
Round 3 participants’ comprehension scores	81.6 (21.8)	58-100	3 (60)
Round 4 participants’ comprehension scores	88.4 (17.5)	58-100	4 (80)

^a^Score is percentage of correct answers out of 12 questions.

#### Feedback on Target Behaviors and Intervention Content

Overall, the participants reported that they found the information useful and easy to understand. They commonly reported already knowing recommended behaviors or that they found the recommended behaviors *common sense* but identified the importance of having the information provided at the right time. They also acknowledged the value of credible sources:

A lot of it seems like common sense...but I suppose, well, now, but maybe in the moment it’s good reminder to have.

I think it’s helpful to have it written as you know, from the doctor’s perspective and I guess it’s quite confronting to hear that so many admissions...are from I guess, avoidable things.

The participants particularly liked that the intervention targeted social and environmental aspects, for example, the importance of rest for the infant’s well-being and the importance of support from family and friends to get enough rest. However, there were some concerns with support not being available for everyone:

It’s telling me that my rest is really important...and um that it’s actually like a safety issue for the baby that I have enough sleep and I just don’t think that that information is out there enough...

Some participants felt that the information is targeted more toward new mothers with 1 baby and pointed out the importance of information being suitable for the broader audience. In addition, they pointed out some information that they believed may not be practical and requested didactic information:

So I guess this app is more targeted towards new mums rather than mums who have already had another baby as well?...if baby is sleeping, we’ll probably be looking after the other one and not really looking after ourselves...

#### Views Toward the App

In general, the participants liked the concept of the app but felt that the delivery of information could be more graphical. They commonly liked the *self-tracking* actions and the idea of receiving reminders and felt that this made the intervention more *app*-like. However, some were confused with the expected use of tracking, that is, as a checklist rather than using it while attending to the baby:

I quite like, and maybe this is just my personality, but I quite like that you can mark as done.

The participants commonly requested additional information related to childcare, which was beyond the scope of the intervention, and some felt that the app scope may be too narrow. They also expressed the importance of the delivery channel if they are to use such an app:

I guess it would be how you would get this app, how much it would cost, is it free or not?...

...scope is too narrow, if you want women to use it. It should be much larger than this. It’s not just about falling and placing, its about why they scream, what the signs are...just like if you want somebody for real to use the app.

Mixed feedback was received about the chat feature, with some having concerns about moderation, bullying, and unsound advice being provided on social media platforms. Others felt that it might be a good place to open up about issues that they cannot raise with their immediate family and requested a professional moderator for the chat. Overall, it was clear that they saw this feature as a place to raise all infant-related questions rather than questions relevant to the intervention:

Yea, I don’t know, I just find it hard to...yea, cos of like Facebook and stuff and there’s a lot of bullying and judgement and what not...I don’t want mums to feel like bullied or that like they’re doing the wrong thing, they’re already so vulnerable.

...you’ve got heaps of apps like that out there already but to have access to someone with medical advice would be amazing.

#### Key Intervention Modifications After User Testing

After review of the feedback, the modifications, as summarized in Textbox 1, will be taken into future app development.

Screenshots of the app are available in Multimedia Appendix 3.

**Feedback, key takeaways, and app modifications.**

**App scope may be too narrow or niche**

- When testing future modules, users will be given a version that will look visually similar to the final app consisting of multiple modules

**Importance of timed information or reminders**

- A feature where users can set up local reminders for actions will be implemented in future modules. The final intervention will include timed push notifications to further support adherence with the actions

**Importance of providing practical advice**

- Special consideration was given to ensuring the practicality of the information and the app features

**Perceived complications with a group chat feature**

- Group chat feature requires modification. A feature to submit questions to a professional has been suggested as a replacement for this feature, and the practicality of this is being investigated

## Discussion

### Principal Findings

This paper describes a behavior theory and user-centered approach to developing a DBCI, an intervention to target the problem of infant falls. In this paper, we have outlined the entire development and user-testing process undertaken to construct an intervention module targeting falls that occur while the infant is feeding. The same process is being applied to 3 more modules targeting the remaining common fall mechanisms: (1) *falls from furniture,* (2) *falls from baby products,* and (3) *falls related to risky home environments (eg, steps and stairs)*; the module used as the case study in this paper was arbitrarily chosen. The decision to present just 1 module as a case study was made to ensure that the full detail of the systematic intervention development method could be presented.

The systematic exploration of the problem from a behavior perspective and the identification of intervention content to specifically target behavior using the strong theoretical base of the Behaviour Change Wheel [7] is a strength of this development process. The need to ground injury prevention interventions targeting behavior in behavior theory has been clearly acknowledged [37], and the Behaviour Change Wheel and the COM-B Model—which proposes that there are 3 components to any behavior (B): capability (C), opportunity (O), and motivation (M)—are increasingly being used for this purpose in other contexts [38,39]. However, there is a relative paucity of studies in the literature describing processes for achieving this, particularly in the context of injury prevention. Similarly, although person-centered approaches to developing DBCIs have been used extensively in other areas of health to produce effective digital interventions [40,41], there seems to be limited application of this type of systematic approach to injury prevention digital intervention development. The work described in this paper fills both gaps.

In our behavior theory–driven approach using the COM-B Model and Behaviour Change Wheel we used a literature review and qualitative analysis of infant fall events from a web-based parenting forum [23] to identify the problem behaviors targeted in this intervention. The research team in consultation with a broader group of experts then selected behavior change functions and techniques. In other contexts, different approaches have been used. For example, others have used stakeholder meetings and interviews with the target audience [39] or surveys [38] to identify target behaviors. The critical similarity in the different approaches is reliance on data collected directly from the target population rather than assumptions from research teams on what behaviors need to change and what might be driving these behaviors.

Another strength is the inclusion of a comprehension assessment in the user-testing component. This is not a common feature of person-centered approaches to behavior change and DBCIs; yet, in other areas of health communication, ensuring comprehension is recognized as critical [25]. This also somewhat addresses the call to pay greater attention to eHealth literacy made in a recent systematic review of digital health interventions for injury prevention [42]. However, in addition to understanding the content of the digital intervention, there is also a need to ensure that users can adequately navigate to *seek and find* information [43]. We intend to assess this in the next phase of development, which will combine the intervention modules within an integrated app and undergo longitudinal testing.

In addition to describing the intervention development process, this paper also demonstrates the benefit of the user-testing process in behavior change app development. Several important insights from user perspectives have been identified that may be important for encouraging the use of the app in parents of infants, and we will attempt to incorporate these strategies in the final integrated app. Of particular interest is the feedback centered around integrating the injury prevention intervention into an app with broader scope and incorporating noninjury prevention advice to mothers and caregivers of infants. Although there is emerging interest in the integration of injury prevention with more general pediatric health care [44,45], to our knowledge there has been little formal investigation of the efficacy of embedding targeting child injury prevention interventions within the context of child and family health care, including general parenting advice. In other contexts, researchers have noted that motivation and engagement with interventions delivered digitally through mobile technologies may be increased by providing features that the user sees as beneficial [46]. This may be a worthy area of further exploration regarding increasing parental engagement in digital injury prevention interventions and, as noted by Issom et al [46], highlights the need for participatory approaches to digital intervention development.

The intervention development process we have described increases the likelihood that the intervention will be effective in promoting desired parental behaviors for preventing infant falls. The process should also increase acceptability and usability of the end product among the target audience. However, the work to date does not yet demonstrate this. Once the intervention modules have been integrated into the app, there will be a need to robustly establish the effectiveness of the intervention. This is particularly important because despite reports of the promise of mobile behavior change interventions for reducing childhood injury [42,47], there are relatively few trials reporting effectiveness of DBCIs targeting childhood injury prevention.

More broadly, our user-centered approach to intervention development and intention to robustly evaluate the effectiveness of the intervention responds to research needs in the digital health care space generally [13,14]. The intervention development process we have described could be applied to many other settings where there is a need for theory- or evidence-informed intervention that relies on user acceptance and engagement.

A limitation observed in the user-testing phase of the study is the homogeneity of the mothers recruited. All were relatively highly educated and from high-income sectors of the community. This is problematic, given that the target audience for this intervention includes the complete demographic range of parents of infants, particularly because it is recognized that there is an increased risk of injury among children from the lower socioeconomic sectors of communities [48]. Previous work has identified that >95% of women in a high-income country setting own a smartphone regardless of individual sociodemographic factors [49], indicating that the bias in our sample reflects a limitation of the study rather than a limitation in the intention of our intervention, that is, using a smartphone digital delivery method. This study limitation highlights the need to use broader recruitment strategies to ensure that women from a wider variety of backgrounds are invited to participate. A potential strategy for achieving this would be to conduct user testing over a broader geographic area that incorporates wider sociodemographic diversity. Similarly, for other injury types, it will be useful to recruit other common carers such as fathers, coparents, and grandparents.

### Conclusions

The work presented in this paper provides a detailed description of a behavior theory–driven and person-centered approach to designing, developing, and optimizing a DBCI targeting a significant childhood injury problem. The process described and the intervention being developed address important gaps in the literature regarding the development of digital child injury prevention interventions. Ultimately, this work represents the first stage in the development of a unique intervention targeting the widespread problem of falls in children aged <1 year. This will be the first intervention of its kind, and as demonstrated in this paper, it is being developed in a unique, systematic, and robust manner.

## Appendix

Multimedia Appendix 1 Think-aloud interview protocol.

Multimedia Appendix 2 Comprehension-assessment questionnaire.

Multimedia Appendix 3 Screenshots of the app.

## Abbreviations

DBCI: digital behavior change intervention
