# Supplementary material for: Toward a Behavior Theory–Informed and User-Centered Mobile App for Parents to Prevent Infant Falls: Development and Usability Study
Source: JMIR Pediatr Parent. 2021 Dec 20;4(4):e29731. doi: 10.2196/29731 (PMC8726019; doi:10.2196/29731)
Supplement: Multimedia Appendix 1 [file pediatrics_v4i4e29731_app1.pdf]

Think aloud interview protocol

Introduction: Thank you for agreeing to be interviewed. As you know we are doing this think aloud interview to get your opinion as a potential future end user of the app that we are developing to prevent infant fall related injuries.

This module that we are testing today will target infant falls often occur during or after feeding.

To test the module, you will be given set of tasks which will make you use the app. For each task you have to,

- Think out loud as you complete tasks
- Describe what you've read/watched in your own words
- Describe what action you would take after reading/watching the content
- How confident you are that you could do what the content is asking

Tasks

1. Complete the "on boarding" section
2. Complete "breast feeding" section
3. Complete "bottle feeding" section
4. Complete "getting enough rest" section
5. Browse the "actions" section
6. Browse the app

General questions

1. What are your overall views toward the app?
2. What do you think about the information provided within the app?
3. What do you think about the aesthetics of the app i.e. colours, text sizes, use of graphics
4. Was there anything you particularly disliked (app feature/information)?
5. Was there anything you particularly liked (app feature/information)?
6. Do you have any suggestions for how the app could be improved?
7. Are there any other comments you would like to make?
