# Supplementary material for: Toward a Behavior Theory–Informed and User-Centered Mobile App for Parents to Prevent Infant Falls: Development and Usability Study
Source: JMIR Pediatr Parent. 2021 Dec 20;4(4):e29731. doi: 10.2196/29731 (PMC8726019; doi:10.2196/29731)
Supplement: Multimedia Appendix 2 [file pediatrics_v4i4e29731_app2.pdf]

Comprehension assessment (for use by researchers)

(Interviewer NB: Tell participant to use the app on their own taking as long as they need)

(Interviewer NB: Ask participant to inform you when they have finished going through the app and begin the following questions)

Total time taken to familiarize with the app :

	Question	Information Required	Information located	Information understood
1	What is the most common cause of infant injury in Australia	Falls		
2	What is the body part most commonly injured due to a fall in infant	Head		
3	What is the most important thing to do to prevent falls while feeding	Getting enough rest		
4	Name two things to do to make sure you will get enough rest with your baby that you can do **before the baby arrives**	Two out of<br>a. Talk to the doctor about baby blues<br>b. Make a sleep plan with partner/grandparents<br>c. Buy a breast pump<br>d. Not taking additional responsibilities		
5	Name three things to do **with your baby** to make sure you get enough rest with your baby	Three out of<br>a. Utilize hospital nursery<br>b. Sleep when the baby sleeps<br>c. Ask for help<br>d. Buy a sling/baby carrier<br>e. Settle into routine<br>f. Take shortcuts around the house<br>g. Seek out sleep specialist		

6	What is the most possible reason for infant falling during breastfeeding	Mother falling asleep while holding the baby		
7	Name the 3-step method to follow during breastfeeding	Prepare position place		
8	What is the best place to breastfeed when you are not tired	Sitting in a comfortable chair		
9	Why it is risky to feed sitting if you are tired	Risk of SIDS and falls		
10	What is more safer place to breastfeed if you are tired	Bed/middle of the bed		
11	What is the best place for the baby to sleep after a feed	Cot		
12	Why you should not leave baby alone on a bed when or after bottle-feeding	Risk of rolling down		
