# Supplementary material for: Toward a Behavior Theory–Informed and User-Centered Mobile App for Parents to Prevent Infant Falls: Development and Usability Study
Source: JMIR Pediatr Parent. 2021 Dec 20;4(4):e29731. doi: 10.2196/29731 (PMC8726019; doi:10.2196/29731)
Supplement: Multimedia Appendix 3 [file pediatrics_v4i4e29731_app3.pdf]

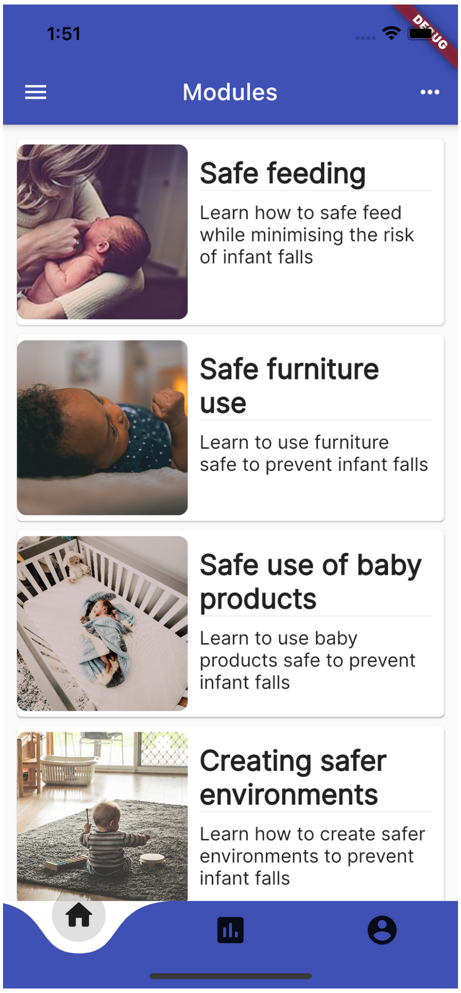

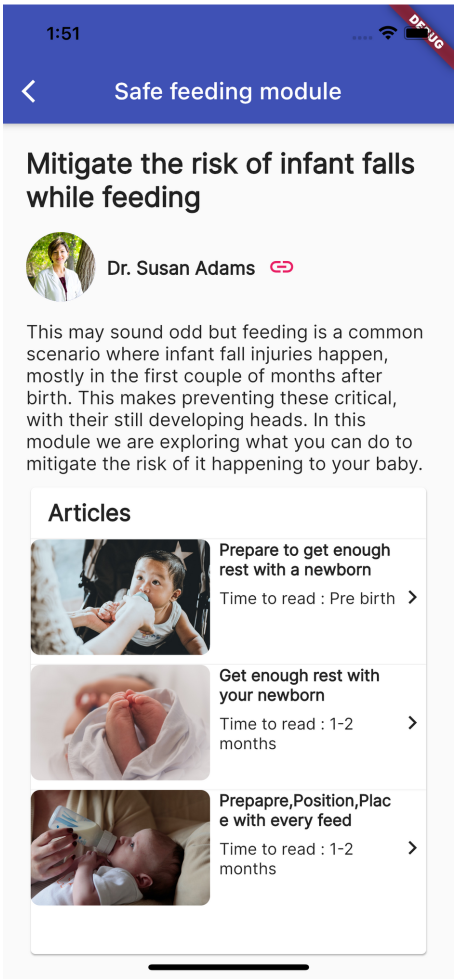

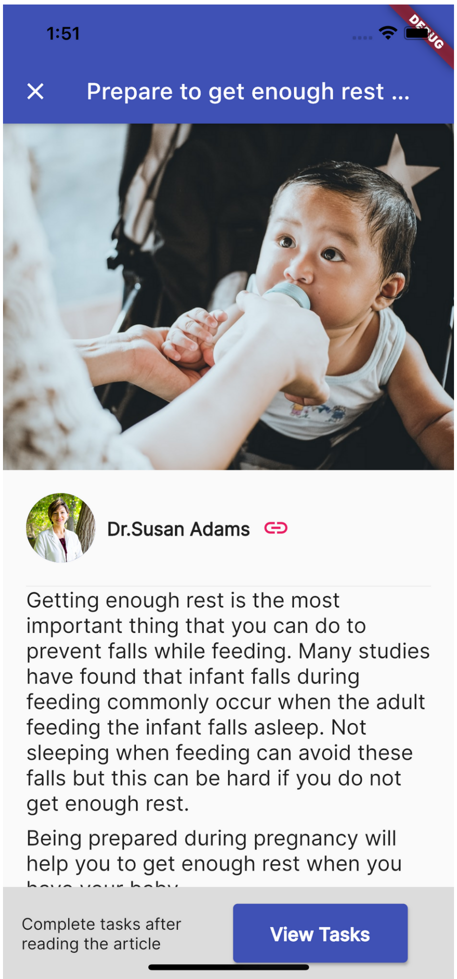

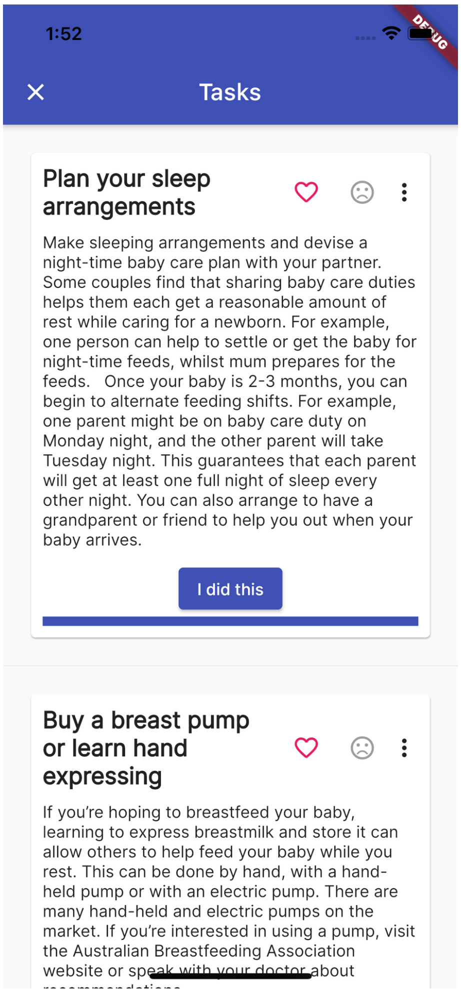

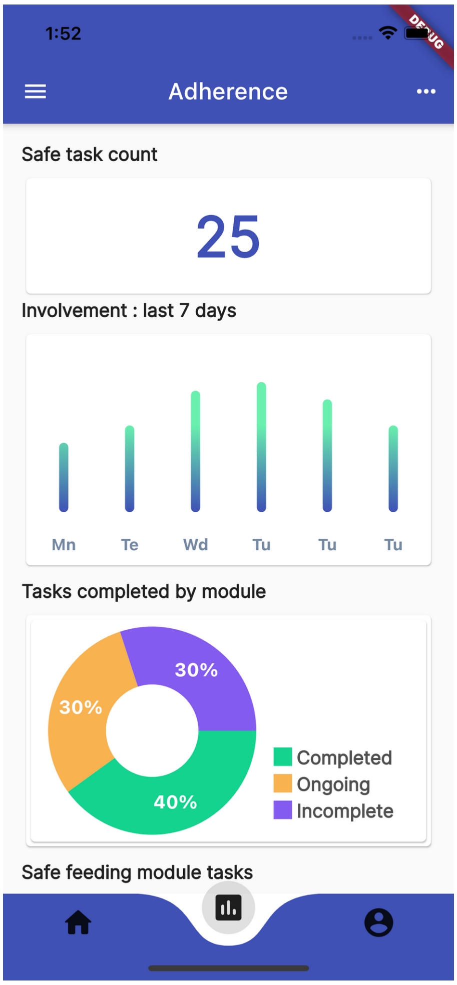

App screens

1. Home screen
2. Module home
3. Articles
4. Actions/tasks screen
5. Task adherence screen

(Note that there are from a beta release, not the final application)
